# Total testosterone and neuropsychiatric symptoms in elderly men with Alzheimer’s disease

**DOI:** 10.1186/s13195-015-0107-4

**Published:** 2015-05-01

**Authors:** James R Hall, April R Wiechmann, Rebecca L Cunningham, Leigh A Johnson, Melissa Edwards, Robert C Barber, Meharvan Singh, Scott Winter, Sid E O’Bryant, for the Texas Alzheimer’s Research and Care Consortium

**Affiliations:** Institute of Aging and Alzheimer’s Disease Research, University of North Texas Health Science Center, 3500 Camp Bowie Blvd., Fort Worth, Texas 76107 USA; Department of Psychiatry and Behavioral Health, University of North Texas Health Science Center, 3500 Camp Bowie Blvd., Fort Worth, Texas 76107 USA; Department of Pharmacology and Neuroscience, University of North Texas Health Science Center, 3500 Camp Bowie Blvd., Fort Worth, Texas 76107 USA; Department of Internal Medicine, University of North Texas Health Science Center, 3500 Camp Bowie Blvd., Fort Worth, Texas 76107 USA

## Abstract

**Introduction:**

There has been a significant increase in the use of testosterone in aging men, but little investigation into its impact on men with Alzheimer’s disease (AD). The findings of the few studies that have been done are inconsistent. In the present study, we investigated the relationship between total testosterone (TT) and neuropsychiatric symptoms (NPS) in a well-characterized sample of elderly men with mild to moderate AD.

**Methods:**

The sample, which was drawn from the Texas Alzheimer’s Research Care Consortium Longitudinal Research Cohort, included 87 men who met the criteria for mild to moderate AD. The occurrence of NPS was gathered from caregivers and/or family members with the Neuropsychiatric Inventory. TT was analyzed, and the sample was divided into a low-testosterone group (TT ≤2.5 ng/ml; n = 44) and a borderline/normal group (TT ≥2.6 ng/ml; n = 43).

**Results:**

TT was correlated with symptoms of hallucinations, delusions, agitation, irritability and motor activity. The borderline/normal group was significantly more likely to have hallucinations (odds ratio (OR) = 5.56), delusions (OR = 3.87), motor activity (OR = 3.13) and irritability (OR = 2.77) than the low-testosterone group. Health status and apolipoprotein E ε4 status were not significant factors.

**Conclusions:**

The findings of the present study have implications for the use of testosterone replacement therapy in men with AD or the prodromal stage of the disease.

## Introduction

The majority of individuals with Alzheimer’s disease (AD) experience one or more neuropsychiatric symptoms (NPS) [1] during the course of the disease. The presence of these symptoms has a significant impact on quality of life, both for the patients [2] and for their caregivers [3,4], and increases the risk of nursing home placement and death [5]. The occurrence of these symptoms in AD is affected by a number of factors, including sex [6], previous psychiatric history [7], stage of the disease [8] and situational factors [9]. In our prior work, we began to identify potential blood-based biomarkers of NPS in AD [10,11]. Sex-specific patterns of biomarkers [10] emerged, with dysregulation of inflammatory biomarkers being related to the occurrence of NPS in women and elevated total cholesterol related to NPS in men [10,11]. Different patterns of inflammatory biomarkers were found for female apolipoprotein E ε4 (APOE ε4) carriers and noncarriers, whereas APOE ε4 status was not a factor for men [11]. These sex differences suggest that sex hormones may be related to the occurrence of NPS in AD.

A number of studies have reported a link between low testosterone levels and the risk for developing AD [12-14]. Testosterone has been proposed to be neuroprotective by affecting amyloid-β protein metabolism and oxidative stress [15]. Low levels of testosterone have been related to poorer sense of psychological well-being [16] and subsyndromal levels of depression and anxiety in healthy older men [17]. High levels of testosterone may have a positive effect but also may be detrimental [18]. Studies of aggression in men have found a relationship between high levels of testosterone and violence and aggression [19,20]. Bufkin and Luttrell [21] reviewed studies of neuroimaging related to emotional and aggressive behaviors and found that the prefrontal, temporal and subcortical substructures of the hypothalamus and amygdala were involved. Batrinos [19], in his discussion of testosterone and aggressive behavior, described the subcortical structures of the hypothalamus and the amygdala as the places where aggression and emotions are “born” and the prefrontal cognitive structures as the places where they are “perceived and controlled.” High levels of testosterone are seen as downregulating the interaction between the cognitive and emotional systems and reducing the effectiveness of cognitive controls [22]. AD has a significant impact on those areas of the brain that are involved in the expression and control of emotional behavior. The possible link between testosterone and aggression and other NPS in AD has not been extensively investigated, and the results of the limited research that has been done tends to be contradictory. Orengo *et al*. [23] found a relationship between testosterone levels and aggression in elderly men with dementia. There was no relationship between testosterone and total score on the Neuropsychiatric Inventory (NPI), a measure of the occurrence and severity of NPS. Xing *et al*. [24] found no relationship between levels of sex hormones, including testosterone, and NPS in a sample of men with vascular dementia, although sex hormones were associated with NPS among women. In a study of the impact of APOE ε4 status on the relationship between sex hormones and NPS in AD [25], sex-specific effects of testosterone were found regarding the occurrence of agitation and/or aggression, but again only for women, not for men.

In the present study, we sought to clarify the link between testosterone and NPS in AD by investigating the relationship of total testosterone (TT) with the occurrence of NPS in a sample of elderly men with AD.

## Methods

### Participants

The sample was drawn from individuals enrolled in the Longitudinal Research Cohort of the Texas Alzheimer’s Research Care Consortium (TARCC) who had a complete serum biomarker panel and a completed NPI interview. The TARCC is a longitudinal multisite cohort of patients with AD and normal controls in which each participant undergoes an annual evaluation that includes a medical examination, an interview, neuropsychological testing and a blood draw. Patients with AD met consensus-based diagnostic criteria for probable AD based on National Institute of Neurological and Communicative Disorders and Stroke and Alzheimer’s Disease and Related Disorders Association guidelines [26]. As we investigated the role of testosterone in elderly men, only men were included in the sample. The final sample consisted of 87 men who met the diagnostic criteria for AD. The mean age of the sample was 75.67 years (standard deviation (SD) = 8.03); the average education level was 13.44 years (SD = 4.32); the mean Mini Mental State Examination (MMSE) score was 20.07 (SD = 6.79); the mean Clinical Dementia Rating (CDR) scale score was 1.10 (SD = 0.73); and the mean CDR Sum of Boxes score was 6.62 (SD = 4.47). The total years of education completed was determined by patient self-report. APOE ε4 carriers made up 54% of the participants, and 96% of the participants were non-Hispanic Caucasians. Institutional review board approval was obtained at each TARCC site (see Acknowledgements for details), and written informed consent was obtained from all participants and/or their caregivers.

As part of the TARCC evaluation, individuals familiar with the behavior of the participants (that is, caregivers and/or family members) who accompanied the participants to the evaluation were administered the NPI Questionnaire, which is a brief, valid and reliable instrument used in the assessment of NPS [27]. When more than one caregiver attended the evaluation, the NPI was conducted with the individual with greatest knowledge of the participant’s behavior. Consistent with our previous research [10,11], the analyses in this study were focused on the presence or absence of a behavior rather than on the more subjective estimate of perceived severity. In the analyses, the occurrence of NPS and the total number of symptoms reported, as well as of each of the specific symptoms, served as the primary outcomes.

### Biomarkers

The TARCC research platform uses the Myriad RBM Human Multi-Analyte Profile multiplexed immunoassay (HumanMAP; Myriad RBM, Austin, Texas, USA) to analyze blood-based biomarkers.

### Assays

Nonfasting samples were drawn with 21- to 23-gauge needles into 10-ml serum-separating (tiger top) vacutainer tubes at the time of interview. Samples were allowed to clot at room temperature for 30 minutes in a vertical position before being centrifuged at 1,300 × *g* for 10 minutes. Next, 1-ml aliquots were pipetted into polypropylene cryovial tubes and placed in −20°C (non-frost-free) or −80°C freezers until shipment to the TARCC Biobank for long-term storage at −80°C. Total processing time (from needlestick to freezer) was 2 hours or less. All samples obtained from the present project were shipped on dry ice to Myriad RBM for assay on the Luminex-based HumanMAP 1.0 platform (Luminex, Austin, Texas, USA).

### Data analysis

TT was determined for each participant. The participants were grouped into hypogonadal or borderline/normal based on the widely accepted clinical practice guideline [28,29] definition of hypogonadism as TT ≤2.5 ng/ml. Participants were grouped into either the low-testosterone group (Low T) (TT ≤2.5 ng/ml) or the borderline/normal testosterone group (Normal T) (TT ≥2.6 ng/ml). The total number of participants with TT ≤2.5 ng/ml was 44, and 43 participants had TT ≥2.6 ng/ml and thus were in the Normal T group. APOE ε4 status (carriers versus noncarriers) was also analyzed. Data were analyzed using product-moment correlations, *t*-tests and multivariate analysis of variance. The 0.05 level of significance was applied to the data.

## Results

Table 1 shows that the sample was composed of older men with a relatively high level of education in the mild stage of disease progression. When divided based on testosterone level, the two groups did not differ with regard to age, years of education or level of cognitive impairment (as assessed by the MMSE and CDR-Global score). Disease progression as assessed by the CDR Sum of Boxes did not differ between the two groups. There was no difference between the groups with regard to percentage of APOE ε4 carriers. The number of total NPS reported for each group was not significantly different. Analysis of the two groups regarding health history showed no significant differences in the percentage of individuals with hypertension, hyperlipidemia, diabetes or obesity. Product-moment correlations (Table 2) revealed significant correlations (*P* < 0.05) between TT and the total number of NPS reported, along with the symptoms of hallucinations, delusions, agitation, irritability and motor activity. Multivariate analysis of variance revealed no significant main effect for TT.

**Table 1:** **Characteristics of the sample** ^**a**^

	**Total sample**	**Low testosterone**	**Borderline/Normal testosterone**	***P*** **-value**
	**(** ***N*** **= 87)**	**(** ***n*** **= 44)**	**(** ***n*** **= 43)**	
Age, yr	75.67 (8.033)	77.24 (7.845)	74.14 (8.008)	0.075
Education level, yr	13.44 (4.324)	14.14 (3.771)	12.74 (4.746)	0.137
MMSE score	20.07 (6.789)	20.45 (5.584)	19.70 (7.839)	0.611
CDR-Global score	1.10 (.727)	1.08 (.698)	1.12 (0.762)	0.836
CDR-SOB score	6.62 (4.474)	6.38 (4.019)	6.86 (4.914)	0.624
NPI total	4.15 (2.888)	3.619 (2.408)	4.674 (3.235)	0.092
Testosterone, ng/ml	2.49 (1.046)	2.11 (.804)	3.94 (0.348)	0.000*
APOE ε4 carriers/noncarriers	54%/46%	53%/47%	54%/46%	0.909
Hyperlipidemia	65%	60%	68%	0.455
Hypertension	64%	68%	61%	0.527
Diabetes	17%	17%	17%	0.963
Obesity	19%	14%	24%	0.239

^a^APOE ε4, Apolipoprotein E ε4; CDR, Clinical Dementia Rating; MMSE, Mini Mental State Examination; NPI, Neuropsychiatric Inventory; SOB, Sum of Boxes. Data presented are mean (standard deviation) or percent values. *Significant *P* < 0.05.

**Table 2:** **Correlations between total testosterone and neuropsychiatric symptoms** ^**a**^

**NPI symptoms**	**Total testosterone (** ***r*** **-value)**	***P*** **-value**
NPI total	0.276	0.009*
Hallucinations	0.242	0.020*
Delusions	0.208	0.040*
Agitation	0.212	0.037*
Irritability	0.319	0.003*
Motor	0.243	0.020*
Elation	−0.022	0.427
Apathy	0.014	0.454
Disinhibition	0.094	0.454
Depression	0.123	0.152
Anxiety	0.079	0.255
Nighttime	0.081	0.248
Appetite	0.129	0.141

^a^NPI, Neuropsychiatric Inventory. *r*-values represent product-moment correlation. *Significant at *P* < 0.05.

Multivariate analysis of variance was carried out to assess the differences between the two testosterone levels on the specific symptom domains of the NPI. The effect of APOE ε4 status was also assessed. The analysis revealed significant differences between the testosterone-level groups on the reported occurrence of hallucinations (*F*(1, 86) = 4.339 (*P* = 0.04)), symptoms of irritability (*F*(1, 86) = 5.747, *P* = 0.022)) and symptoms of motor activity (*F*(1, 86) = 5.708, *P* = 0.019)). No significant differences between the two groups were found for any of the other symptoms assessed by using the NPI. APOE ε4 status was not a significant factor, and no significant difference was found between the groups for frequency of APOE ε4 carriers versus noncarriers.

Odds ratios (ORs) were calculated for the NPI symptoms (Table 3) by comparing the two groups based on testosterone level. The likelihood of having hallucinations reported was 13 times greater for the Normal T group. Hallucinations were reported infrequently overall, with 1 of the 44 Low T individuals reporting hallucinations and 10 of the 43 Normal T individuals reporting them. Delusions were almost four times (OR = 3.87, 95% confidence interval (CI): 1.137 to 13.177; *P* = 0.022) more likely in the Normal T group, with twelve individuals reporting delusions compared with four in the Low T group. Irritability, which on the NPI is related to being “cranky, impatient or having difficulties dealing with delays or waiting,” was a relatively frequently reported symptom, with 20 members of the Low T group and 30 of the Normal T group being described as having irritability. Irritability was reported nearly three times (OR = 2.77; 95% CI: 1.148 to 6.681, *P* = 0.018) more often in the Normal T group than in the Low T group. Aberrant motor behavior, described on the NPI as engaging in “repetitive activities, such as pacing, handling buttons, or doing things repeatedly,” was reported almost three times (OR = 2.942, 95% CI: 1.104 to 7.841, *P* = 0.024) as often for the Normal T group, with eight individuals in the Low T group and seventeen in the Normal T group having symptoms related to motor disturbances. The ORs for the remaining NPS were not significant.

**Table 3:** **Odds ratios for occurrence of neuropsychiatric symptoms among borderline/normal compared with low-testosterone groups** ^**a**^

**NPS**	**Odds ratio**	**95% confidence interval**	***P*** **-value**
Hallucinations	13.03	1.588 to 106.954	0.003*
Aberrant motor behavior	2.94	1.191 to 7.841	0.024*
Irritability	2.77	1.148 to 6.681	0.019*
Anxiety	1.24	0.552 to 2.986	0.374
Disinhibition	1.82	0.779 to 4.271	0.119
Delusions	3.87	1.137 to 13.177	0.022*
Agitation	1.83	0.779 to 4.271	0.119
Depression	1.26	0.532 to 2.986	0.380
Apathy	1.17	0.493 to 2.767	0.447
Elation	1.03	0.275 to 3.833	0.616
Appetite	1.52	0.645 to 3.585	0.229
Nighttime	1.16	0.496 to 2.703	0.451

^a^NPS, Neuropsychiatric symptoms. *Significant at *P* < 0.05.

## Discussion

There has been a significant increase in the use of testosterone replacement therapy (TRT) for older men [30], without clear evidence of the impact of testosterone on behavior in diseases where cognitive functioning has been compromised. The presently reported research shows a link between testosterone levels and the occurrence of specific NPS in elderly men with AD. This relationship is with symptoms that could be described as “active,” or “acting out” or in the terms used to describe symptoms of schizophrenia “positive” symptoms. No relationship was found for depression, apathy or disturbances of sleep or appetite. Individuals with borderline to normal testosterone levels were significantly more likely to have hallucinations, delusions, aberrant motor symptoms and irritability than those with low testosterone. It could be argued that those with low testosterone would be less likely to have the energy to engage in acting out. Our sample represented a relatively healthy community-dwelling population, and there was no difference on any of the health measures. Frailty and loss of vigor that may accompany low testosterone cannot account for the differences in hallucinations and delusions.

A number of mechanisms have been proposed to explain testosterone’s neuroprotective effects related to the risk of developing AD [31], but there is a lack of explanatory paradigms to explain our findings. The pathological changes that occur in AD, specifically degeneration in the hippocampus, may make the brain more susceptible to the effects of even normal levels of testosterone. The hippocampus has connections with the hypothalamus and amygdala, structures that are involved in emotional reactivity and the inhibition of emotional behavior [32] through projections to autonomic and endocrine emotion generation systems [33]. The salience network, which has connections to these structures, is said to be involved in reactivity to emotional stimuli. Balthazar *et al*. [34] argued that changes in salience network connectivity are related to hyperactivity symptoms in AD. In essence, the individual becomes more reactive to affective stimuli and less discerning. The structures involved in this process have a high density of sex hormone receptor, which are responsive to testosterone [35]. The salience network may also be involved in the formation of delusions. In research on patients with psychosis, the encoding of usually irrelevant stimuli versus relevant stimuli is affected [36] and the irrelevant stimuli take on greater salience. Along with affecting reactivity, testosterone decreases subcortical–cortical functional connectivity [37], reducing the inhibition of emotions by higher cortical structures. Testosterone reduces the regulatory control of the orbitofrontal cortex over the amygdala. Mega *et al*. [38] found lower perfusion in the frontal lobes and related subcortical structures in patients with AD who manifested delusions and hallucinations. Taken together, this research suggests that testosterone may interact with the ongoing changes in the brain due to AD and affect the occurrence of NPS.

There are a number of factors that limit the generalizability of the results of the present study. The sample, although drawn from a well-characterized AD cohort, is small, and similar findings in much larger samples are needed to support our findings. Additionally, the study is cross-sectional and as such assesses the relationship in mild to moderate AD cases at one point in time. Longitudinal research is especially important in these patients, given the age-related changes in testosterone that occur, as well as the changes in the prevalence of the various NPS over time. The sample was overwhelmingly made up of non-Hispanic Caucasians, which further limits the generalizability of the results. TT may not be the best estimate of the level of testosterone directly affecting the brain [39]. Importantly, there was no control over the time of day when the samples were drawn, and testosterone levels are known to fluctuate throughout the day. Additionally, data were not available on the use of testosterone supplementation or other medications that may have had an impact on testosterone levels. An additional limitation relates to the use of informant data. The data on the presence of the NPS was dependent on caregiver reports, and, although the data were gathered by trained interviewers, the interpretation of the symptoms may have varied from informant to informant. Further research with larger samples studied over the course of the disease will be useful to clarify the relationship between testosterone and NPS in AD.

The findings of the present study are suggestive that testosterone level may play a role in the occurrence of “acting out” symptoms. As shown in earlier research on testosterone and cognition [18,40], testosterone may have detrimental rather than beneficial effects in certain circumstances. It may be that once the disease pathology has reached a certain point, whatever neuroprotective effects testosterone may have had no longer exist, and the level of testosterone affects the likelihood of specific negative behaviors.

There are a number of implications of our findings. The strategy of using TRT to improve mood in hypogonadal men with AD may have unintended consequences, and therefore TRT should be used judiciously. Assessing cognitive function may be necessary to aid in determining whether to use TRT in older men who may have unidentified prodromal AD. Additionally, measuring testosterone in older men with AD may aid in identifying individuals who have a greater likelihood of developing NPS as the disease progresses.

## Conclusions

This cross-sectional research on a sample of men with mild to moderate AD found that the men with normal levels of TT had significantly higher frequency of the NPSs of hallucinations, delusions, motor activity and irritability than men classified as hypogonadal. These findings, if supported by further research, have significant implications for the use of TRT in AD and suggest a mechanism by which to identify those at higher risk for developing NPS.

## Abbreviations

AD: Alzheimer’s disease
APOE ε4: Apolipoprotein E ε4
CDR: Clinical Dementia Rating
CDR-SOB: Clinical Dementia Rating Scale Sum of Boxes
CI: Confidence interval
Low T: Low-testosterone group
MMSE: Mini Mental State Examination
Normal T: Borderline/normal testosterone group
NPS: Neuropsychiatric symptom
OR: Odds ratio
SD: Standard deviation
TARCC: Texas Alzheimer’s Research and Care Consortium
TRT: Testosterone replacement therapy
TT: Total testosterone
